# Drug target ranking for glioblastoma multiforme

**DOI:** 10.1186/s42490-021-00052-w

**Published:** 2021-04-26

**Authors:** Radhika Saraf, Shaghayegh Agah, Aniruddha Datta, Xiaoqian Jiang

**Affiliations:** 1 Department of Electrical and Computer Engineering, Texas A&M University, College Station, US; 2 University of Texas Health Science Center at Houston, School of Biomedical Informatics, Houston, US

**Keywords:** Cancer, Boolean network modeling, Drug target ranking, Drug repurposing, Glioblastoma, Drug resistance

## Abstract

**Background:**

Glioblastoma Multiforme, an aggressive primary brain tumor, has a poor prognosis and no effective standard of care treatments. Most patients undergoing radiotherapy, along with Temozolomide chemotherapy, develop resistance to the drug, and recurrence of the tumor is a common issue after the treatment. We propose to model the pathways active in Glioblastoma using Boolean network techniques. The network captures the genetic interactions and possible mutations that are involved in the development of the brain tumor. The model is used to predict the theoretical efficacies of drugs for the treatment of cancer.

**Results:**

We use the Boolean network to rank the critical intervention points in the pathway to predict an effective therapeutic strategy for Glioblastoma. Drug repurposing helps to identify non-cancer drugs that could be effective in cancer treatment. We predict the effectiveness of drug combinations of anti-cancer and non-cancer drugs for Glioblastoma.

**Conclusions:**

Given the genetic profile of a GBM tumor, the Boolean model can predict the most effective targets for treatment. We also identified two-drug combinations that could be more effective in killing GBM cells than conventional chemotherapeutic agents. The non-cancer drug Aspirin could potentially increase the cytotoxicity of TMZ in GBM patients.

**Supplementary Information:**

The online version contains supplementary material available at (10.1186/s42490-021-00052-w).

## Background

Glioblastoma Multiforme (GBM) is the most aggressive primary brain tumor with median overall survival (OS) of 14.6 months to 20.9 months in clinical trial setting and 11 months in all GBM population [1, 2]. Current standard of care (SOC) treatments for GBM include maximum safe surgical resection, radiation, temozolomide (TMZ) chemotherapy and recently FDA approved tumor treating fields (Optune) for newly diagnosed patients as well as bevacizumab (Avastin) for recurrent disease [2, 3]. However, GBM still stays as one of the most challenging cancers to treat due to its complexity or tumor heterogeneity, infiltrative nature and low efficacy of current treatment modalities which results in short-term survival rate. Therefore, novel approaches in the field of GBM drug discovery are needed to overcome current challenges in medication results [4].

A few of the main challenges to GBM treatment are resistance to temozolomide and recurrence of cancer after radiation therapy. Understanding the genetic causes of this resistance to temozolomide is essential while designing therapies to robustly kill GBM tumor cells [5]. Studying the genetic makeup of GBM tumors is also essential while choosing drug combinations for post-radiation chemotherapy. By prioritizing the key genetic targets involved in the progression of GBM, the best drug combination can be predicted.

Using a Boolean network approach to model the gene regulatory networks, we design an application that will predict the best drug combination to treat tumors given their genetic profile.

### Current state of drug discovery

Drug discovery requires the application of different conceptual and analytical approaches to biological processes. The development of a new drug involves identifying new targets, validating said targets, biological synthesis of drugs, considering the pharmacokinetics, studying the potential side effects of the drug, testing, and clinical trials. This process incurs high costs and does not promise great success rates [6]. Recent research shows that even non-cancer drugs can be repurposed to treat cancer; this can offset costs and expand the therapeutic options. The functional testing of all candidate genetic targets or candidate drug combinations becomes infeasible as the number of candidates increases [7].

Molecular and cell biologists are responsible for identifying and evaluating potential targets in the early stages of drug development. The traditional method of ranking drug targets depends on an extensive literature survey of current research and treatment and the knowledge of the researcher. The mental integration of data from a variety of sources can prove to be challenging and is vulnerable to human error. Once a potential target is identified, it needs to be validated through biological experimentation. This trial-and-error method can prove too expensive in terms of resources and time; limitations in the budget and accessibility to appropriate testing facilities can also prove to be obstacles [8].

On the other hand, the newer methods of prioritization of drug targets require access to ample amounts of data and are computationally expensive. High throughput data techniques usually produce only one type of “-omic’ data (genomic, proteomic, metabolomic). Data-based modeling using such data requires specific or proprietary data processing and analysis platforms. The correlative nature of this data makes it challenging to study the exact causal relationships between different data points. Many genes or proteins can have dual roles in biological processes, such as the overexpression of STAT3 in several cancer cells. It might not be possible to determine through such ”-omic” approaches whether STAT3 upregulation is the cause or effect of cancer progression [8–10]. Moreover, the results of the computational models are not easy to interpret and can even conflict with other large-scale ranking techniques. A significant drawback of such approaches is low experimental reproducibility, which means that the ranking is subject to change each time the algorithm is run [7].

We can take a closer look at the latest computational models that predict the drug target ranking. Project Score [11] seems to be a promising prioritization technique, it uses a cellular fitness score to rank targets, and the data is collected using CRISPR/Cas9 screens. The potential demerit of Project Score is that it is not tailored to a specific cancer, and it fails to represent all the cell line mutations found in GBM. DrugComb [7] is a web-based portal that performs large-scale integration of cancer drug screen data for different cell lines; however, DrugComb deals with the drugs as a whole and doesn’t provide information of how individual genetic targets in the GBM pathways could be ranked. The Genomics of Drug Sensitivity in Cancer (GDSC) [12] database includes information about drug sensitivity for different cell lines as well as molecular markers of drug response. The GDSC database considers only anti-cancer drugs. However, while designing optimal cancer therapies, it makes sense to also include the targets of non-cancer drugs [6].

Boolean network modeling offers a tradeoff between data-based modeling and traditional biological methods. Boolean networks are deterministic models that are based on established biological knowledge and can be used to ease the computational burden of the researcher. They are representative of the current state of information that is available for Glioblastoma and can be updated with ease to reflect the latest research–unlike data-based techniques, regenerating the ranking after modification does not require substantial computational power. Our modeling technique seeks to bridge the gap between designing computational models and understanding the biological complexities of cancer. We include existing genetic information as well as research about chemotherapeutic, herbal, and non-cancer drugs. We seek to propose an optimal and robust strategy to combat cancer.

In this work, a Boolean framework has been developed to predict the ranking of combinations of targets in the GBM pathways; this ranking is done on the basis of the ability of the target to induce GBM cell death and to curb cancer cell proliferation. We used this methodology here to develop a new tool for target-based drug discovery.

## Methods

### Biological pathways in glioblastoma

To support the design of targeted therapy for GBM, it is necessary to model the cell signaling pathways involved in the development of cancer. The biological pathways responsible for cell survival and proliferation are dysfunctional in GBM. Genetic aberrations in the cell cycle, such as those originating from mutations in CDK2NA, p53, PTEN, and EGFR, are commonly found in GBM cell lines [13]. Additionally, genes associated with the Fas pathway that is responsible for extrinsic apoptosis (or cell death), are also a feature of GBM tumors. Isocitrate dehydrogenase (IDH) mutations can determine the prognosis of a GBM patient, and the involvement of IDH means the involvement of hypoxia-related and anaerobic metabolic pathways [13, 14]. Finally, the resistance to TMZ can be attributed to the DNA repair pathway, which governs the methylation of Methylguanine-DNA Methyltransferase (MGMT); this motivates the investigation of DNA methylation and histone deacetylation pathways [15, 16]. The model represents the interconnection between these pathways and the other pathways known to be active in cancer cells like the calcium signaling, endoplasmic reticulum stress-related, and stemness (Wnt, Hedgehog, and Notch) pathways [17–19].

Our objective is to show the effect of a gene on cancer cell fate. If a particular target gene or drug is said to be effective in terms of cancer treatment, it should have at least one of the following qualities :

- it should robustly kill cancer cells.
- it should stymie cancer cell growth or proliferation.
- it should abate tumor invasion and metastasis.
- it should curb tumor angiogenesis.
- it should attack the tumor-initiating cells.

To study the effect of the various genes on cancer cell fate, we include the pathways responsible for angiogenesis, inflammation and mitochondrial apoptosis (or cell death).

### Boolean network modeling

The GBM cellular signaling pathways and drug interactions with this network are modeled using the Boolean network approach that has been used in our previous works [20, 21]. Boolean networks are a class of models that can capture the relevant behavior of biological systems and be used for therapeutic purposes. The Boolean network model for different cancers will be distinct; the genetic interconnections for a particular type of cancer vary based on the pathways involved in development of the cancer. For example, the Boolean network model for melanoma [20] does not include the stemness pathways because the stemness genes are not known to play a major role in tumor growth in the skin cancer.

**Fig. 1:**
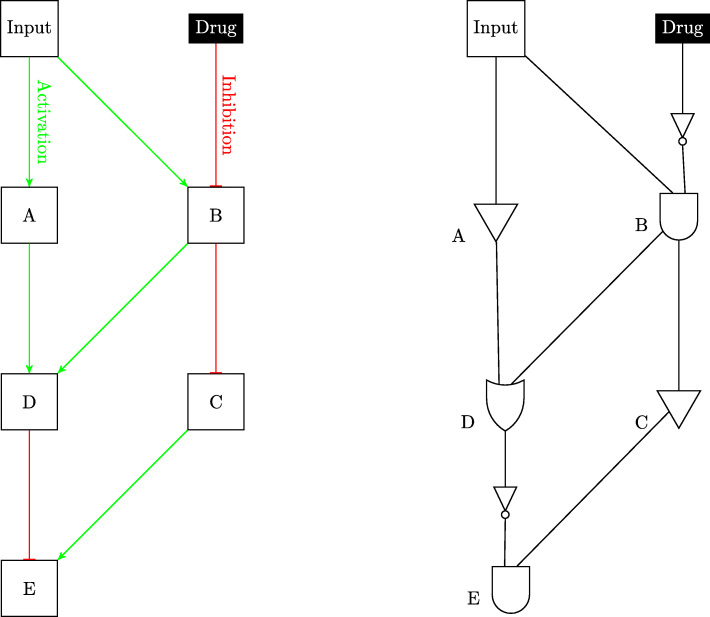
Boolean representation of genetic regulatory networks. LaTeX package tikz version 3.14 is used is used to render this figure

A Boolean network model has three main elements: nodes, edges and inputs, and three main logical functions: AND, OR, and NOT. Each node is either a gene or a receptor and can take only binary values, namely logical 1 and logical 0. If a gene is expressed or up-regulated, it would be in the ON state and quantified in the model by the numeric value 1. If the gene is not expressed or down-regulated, its node would be in the OFF state or quantified by the numeric value zero. Every edge represents an interaction between the nodes of the network. Edges reveal the regulatory relationship between genes, such as inhibition or activation. These relationships can comprise of a single logical function or a combination of logical functions. Drugs, growth factors, cytokines, and other extracellular stimuli are considered as inputs to this system. If any of these inputs are incident on a particular node, then it takes the value of one. Otherwise, the node is set at a value of zero. Inputs can alter the behavior of nodes through either activation or inhibition of a set of target genes. Figure 1 shows how the genetic interactions in pathways are represented using Boolean logic gates.

**Fig. 2:**
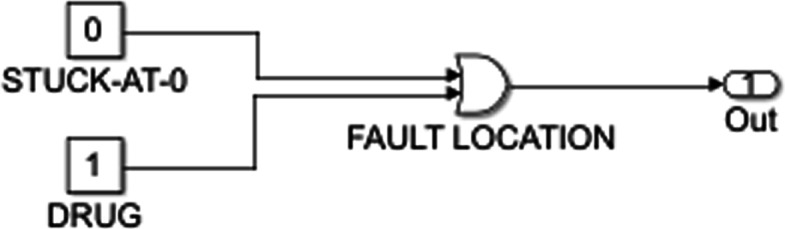
Stuck-at-0 fault. LaTeX package tikz version 3.14 is used is used to render this figure

**Fig. 3:**
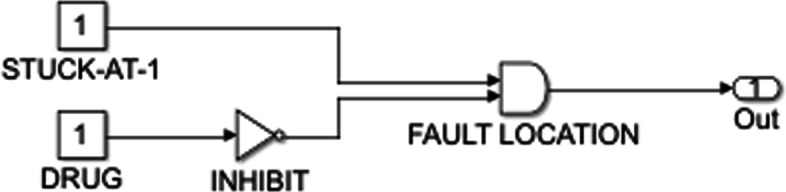
Stuck-at-1 fault. LaTeX package tikz version 3.14 is used is used to render this figure

In order to model a drug in a Boolean network approach, we need to know the targets and the methods of action of the drug. The drug is an input that activates or inhibits its genetic target. Note that one drug can act on multiple targets and can simultaneously activate one target while inhibiting another. If a drug input is ON, then it activates its target through an OR action and inhibits its target through the NOT gate; the two types of actions can be seen in Figs. 2 and 3.

Stuck-at faults are introduced to the system to model genetic abnormalities. Irregularities in the behavior of a gene can be caused by genetic mutation and can potentially lead to cancer. Cancer cellular pathways have different proliferation and apoptosis behavior than normal cellular signaling. A stuck-at fault is a node that remains stuck at a particular value regardless of the value of the input it receives. Genes are at a stuck-at-0 fault if they are permanently deactivated. As can be seen in Fig. 2, a stuck-at-0 fault sets the value of the gene at 0, and the drug corrects this by setting it back to its original value. Similarly, the stuck-at-1 fault occurs if a gene is always in the active state. In Fig. 3, the stuck-at-1 fault forces the gene to take on a value of one, whereas the drug stops this and restores normal function to the node.

Finally, outputs are nodes that are representative of cancer cell fate. These are nodes within the Boolean circuit whose value is measured to test the effect of input on the circuit’s behavior. We will combine the values of these output nodes into one output metric that represents the theoretical efficacy of the drug or target. The theoretical efficacy will consider the anti-cancer ability of the drug or target to drive the circuit to a desirable value, mainly with respect to the ability to induce cell death or stop cell proliferation.

### Prioritization of genetic targets

Protein or mRNA modulation techniques are employed during target validation, where the target is altered by an external agent, and the change in the cellular viability is measured [9]. Cellular viability is the measure of live, healthy cells in the population of cells under experiment, and is inversely proportional to the efficacy of the genetic target.

As an alternative to protein or RNA modulation, we simulate the modulation of the genetic target and measure the change in the output metric. Assume that the Boolean circuit has *N* nodes numbered from 1 to *N*. In each run of the simulation, we force a particular node in the circuit to one, which is equivalent to inducing expression of the corresponding gene, and we measure the change in the output. We will perform *N* runs with node *i*={*True* ∀ *i*∈[1,*N*]}. Similarly, we follow the same steps by forcing every node to zero and measuring the effect of inhibition of one gene at a time. We will perform *N* runs with node *i*={*False* ∀ *i*∈[1,*N*]}. As a result, we have a list of 2*N* measurements of the output metric, each corresponding to a particular Boolean combination in the network. Sorting the list on the basis of the output metric gives us the key intervention points. The most potent intervention point has the maximum effect on the output metric. This is useful while developing single-target therapies.

This technique can be extended to measure the effect of modulating more than one target at a time. We can simulate the effect of altering combinations of targets by forcing groups of nodes to a set of logical values in every run. This is useful while developing multiple-target therapies.

This method can also resolve the controversial dual roles of certain genes. First, we force the node of interest to one (expression) and note the value of the output metric. Next, we force the same node to zero (inhibition) and compare the result to the previous observation. For instance, a comparison of the two values will tell us whether inhibition or expression of that particular gene should stop the progression of the GBM tumor.

We simulate protein and mRNA modulation of various genetic targets in Glioblastoma to isolate the best possible drug combination for treatment of that cancer.

## Results

The static Boolean network in this work maps a set of inputs to a set of outputs; the input and output vectors are given in Eqs. (1) and (2) below. The inputs to the Boolean network are the growth factors, cytokines and extracellular stimuli relevant to the development of GBM. A change in input can cause a change in the output metric. The outputs are a mixture of apoptosis factors as seen in Eq. (3) and genes involved in the cell cycle arrest shown in Eq. (4). For all vectors, i.e input, output, fault and drug vectors, a one in the *i*^*th*^ column of the vector implies that the *i*^*th*^ element is active.

1 $$\begin{array}{@{}rcl@{}}  \text{Inputs}& =& [\text{Shh, Wnt, GF, IL17, Cytokine, TNF, PSEN, TGFb, }\\  & &\text{S1P, Antigen, Dopamine, GABA, Ach, HT, PGE2, EDN1,}\\  &&\text{Norepinephrine, F2, Estrogen, Testosterone,} \\&&{Progesterone, NF1}] \end{array} $$

The Tables 1 and 2 shows the classification of the apoptotic and arrest factors respectively. The fate of the cell depends on the value of these apoptotic and arrest factors.

2 $$\begin{array}{*{20}l} \text{Outputs}& = [\mathrm{Apoptotic\; Factors,\; Arrest\; Factors}] \end{array} $$

**Table 1:** Apoptotic factors

Pro-apoptotic factors	Anti-apoptotic factors
BAK/BAX	BCL2
BID	BCLxL
NOXA	MCL1
PUMA	XIAP
CASP12	CFLIP
CASP8	TERT
DNADamage

**Table 2:** Arrest factors

Pro-Arrest factors	Anti-Arrest factors
DNADamage	HDAC
CHK1	CDK4
	CCND1
	AR

3 $$\begin{array}{*{20}l} \mathrm{Apoptotic\; Factors}& = [\mathrm{BAK, BAX, BID, NOXA, PUMA,}\\ &&\mathrm{CASP12, CASP8, DNADamage}] \end{array} $$

4 $$\begin{array}{*{20}l} \mathrm{Arrest\; Factors}& = [\mathrm{DNADamage,CHK1,HDAC,}\\&&{CDK4,CCND1,AR}] \end{array} $$

**Fig. 4:**
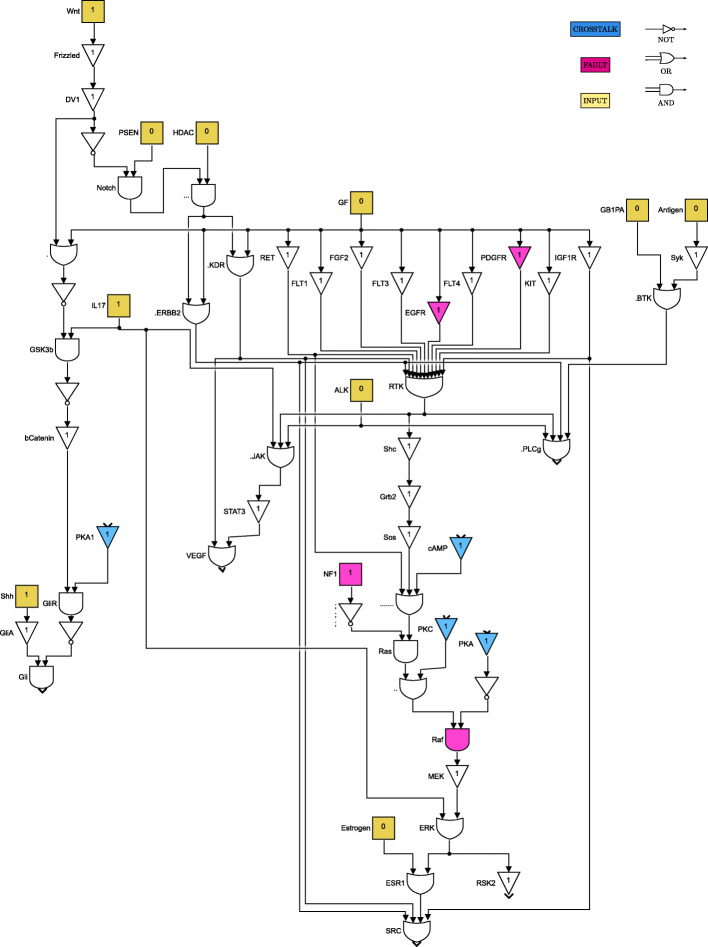
Cell growth and stemness pathways. Simulink version R2020 is used is used to render this figure

**Fig. 5:**
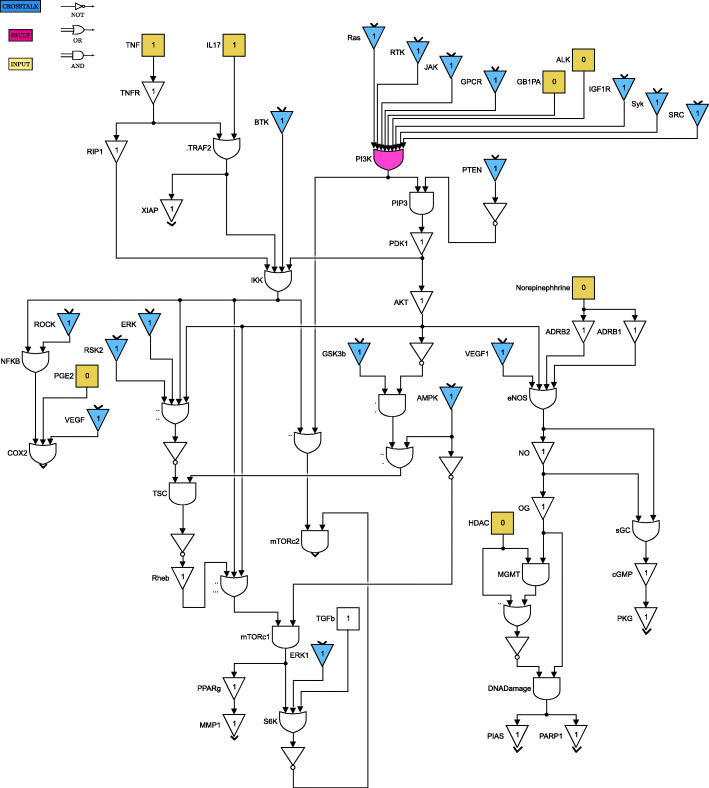
Cell survival, inflammation and histone deacetylation pathways. Simulink version R2020 is used is used to render this figure

**Fig. 6:**
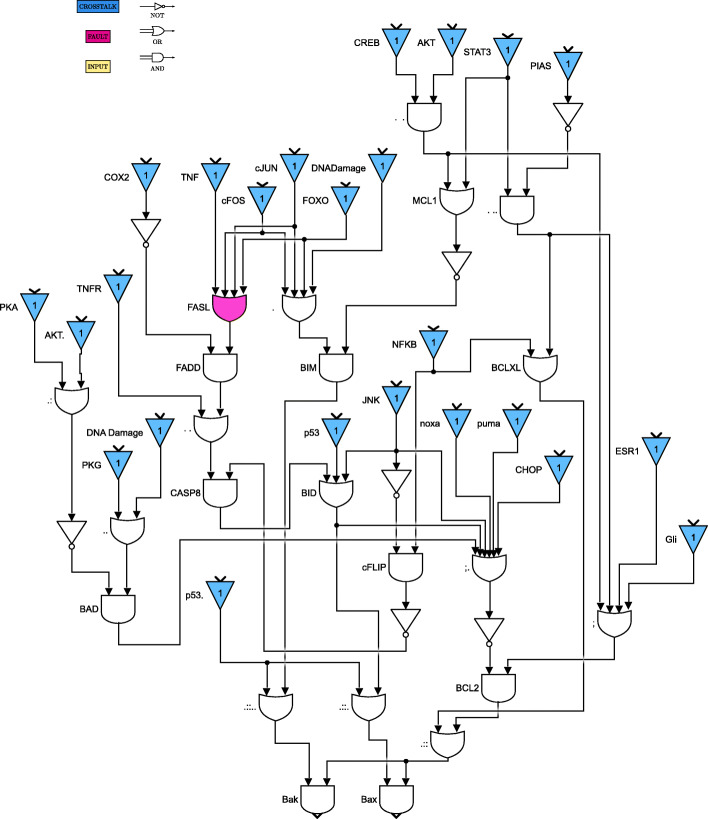
Apoptosis pathways. Simulink version R2020 is used is used to render this figure

**Fig. 7:**
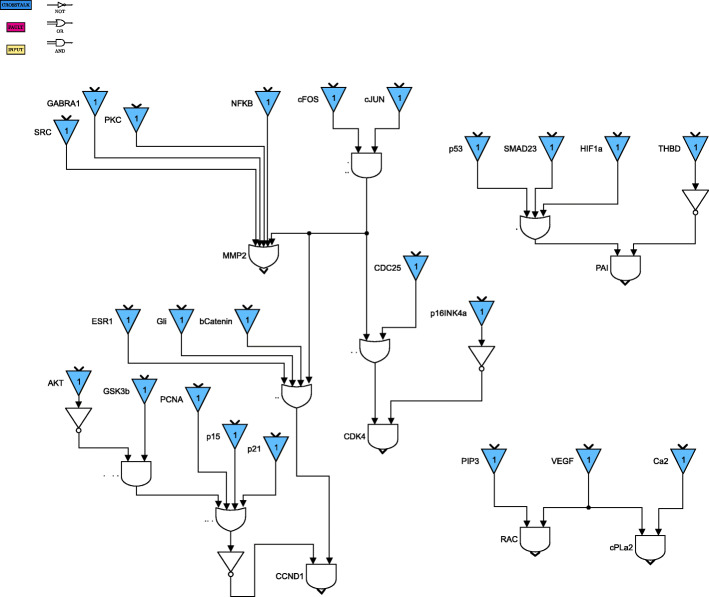
Cell cycle arrest and angiogenesis pathways. Simulink version R2020 is used is used to render this figure

**Fig. 8:**
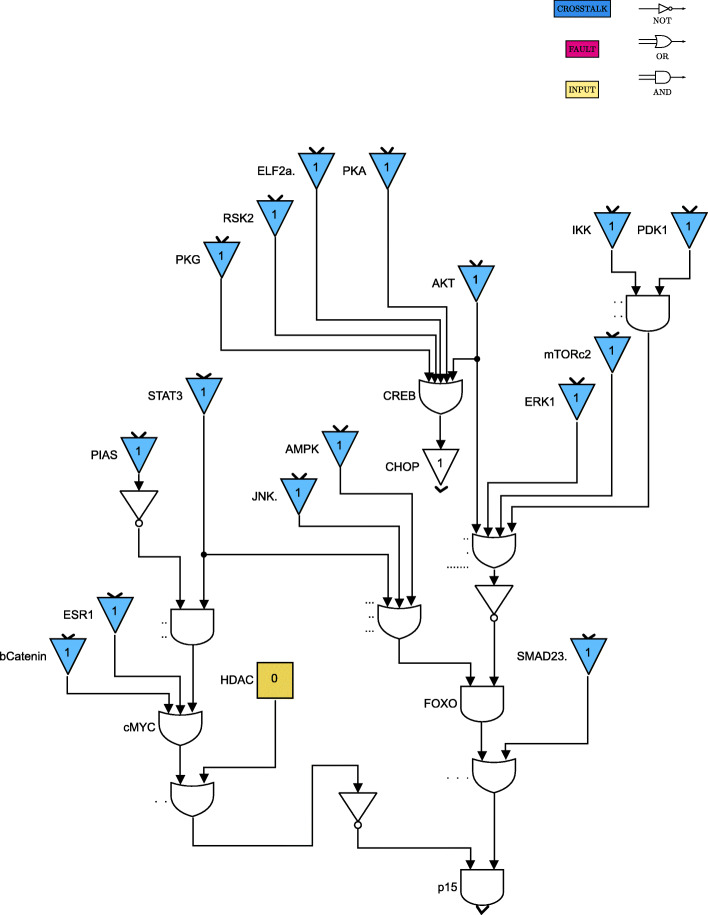
Cell proliferation pathways. Simulink version R2020 is used is used to render this figure

**Fig. 9:**
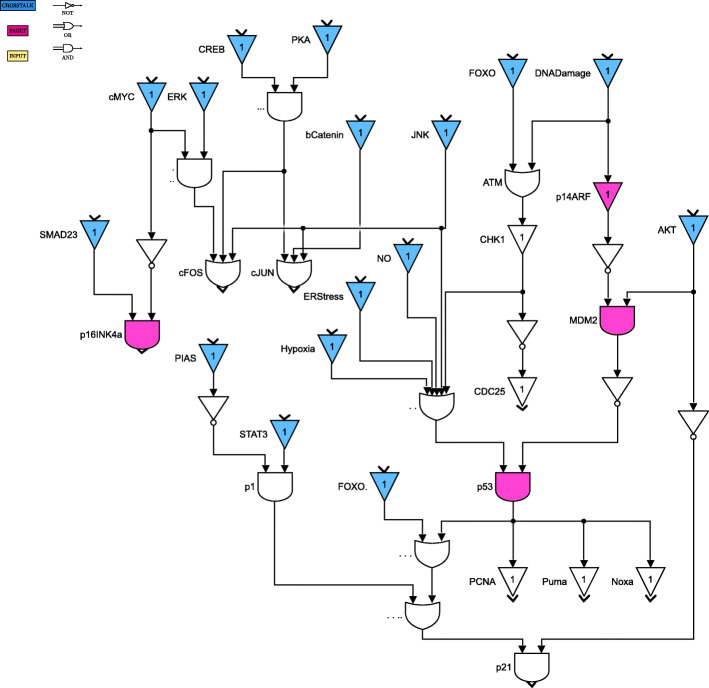
DNA damage and repair pathways. Simulink version R2020 is used is used to render this figure

**Fig. 10:**
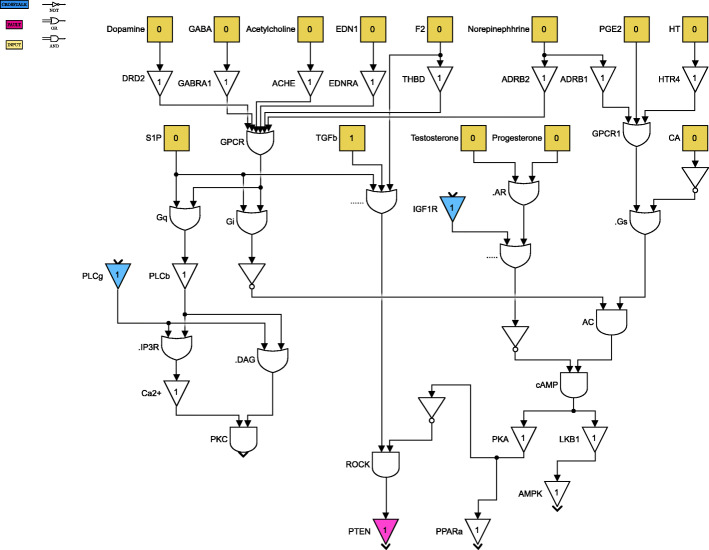
G-coupled protein and calcium signaling pathways. Simulink version R2020 is used is used to render this figure

**Fig. 11:**
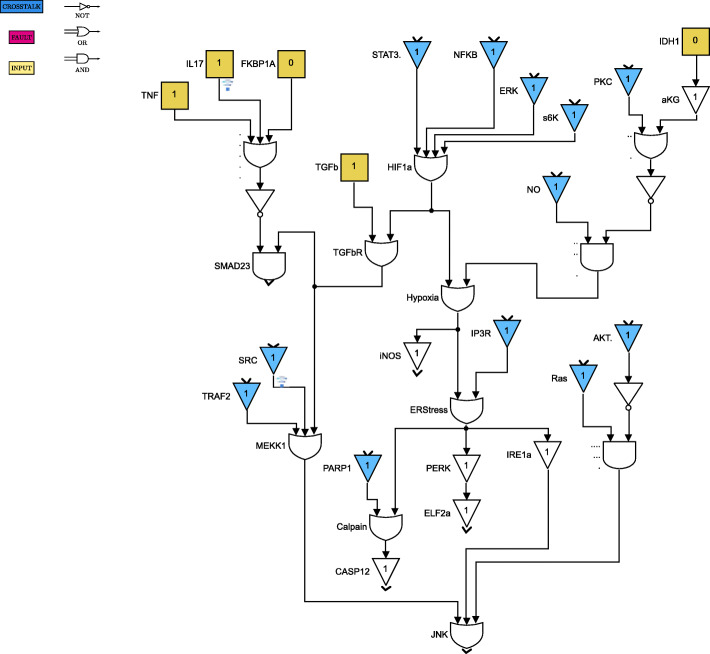
Hypoxia and endoplasmic reticulum stress pathways. Simulink version R2020 is used is used to render this figure

For clarity of exposition, the Boolean network is divided into 8 parts as shown in Figs. 4 through 11. The yellow blocks in the figures represent the inputs to the cells, the magenta blocks represent the genetic mutations commonly found in GBM cell lines and the blue blocks represent the interconnections between the different pathways. Figure 4 shows the cell growth pathways and their cross talk with the stemness pathways namely Wnt- *β*Catenin, Hedgehog and Notch. The histone deacetylation pathway and its interaction with PI3K/mTOR and inflammation pathways is shown in Fig. 5. The output factors that control cell proliferation, cell cycle arrest, angiogenesis and cell death are found in Figs. 6, 7 and 8 respectively. The DNA damage and repair network in Fig. 9 captures the commonly occurring faults in GBM. Figure 10 shows how the g-coupled protein receptors influence calcium signaling and cAMP-PKA pathway in the brain. The hypoxia and endoplasmic reticulum stress-related pathways are active in several cancers including GBM and are shown in Fig. 11.

### Output metric

The output factors have been combined into one output metric to calculate the efficacy of the drug or target. The metric used is given in Eq. (7) and is a combination of the apoptosis ratio in Eq. (5) and the arrest ratio in Eq. (6).

5 $$\begin{array}{*{20}l} \text{Apoptosis Ratio} &= \frac{\sum \text{Pro-Apoptotic factors}}{\sum \text{Anti-Apoptotic factors}} = R_{apo} \end{array} $$

6 $$\begin{array}{*{20}l} \text{Arrest Ratio} &= \frac{\sum \text{Pro-Arrest factors}}{\sum \text{Anti-Arrest factors}} = R_{arr} \end{array} $$

7 $$\begin{array}{*{20}l} \text{Theoretical Efficacy} &= \frac{N_{apo}}{N} R_{apo}+ \frac{N_{arr}}{N}R_{arr} \end{array} $$

The apoptosis ratio *R*_*apo*_ denotes the relative change in cell death for each different set of inputs and *N*_*apo*_ is the number of pro-apoptotic factors and anti-apoptotic factors in total. The arrest ratio *R*_*arr*_ denotes the relative change in cell cycle progression for each different set of inputs and *N*_*arr*_ is the number of pro-arrest factors and anti-arrest factors in total. Finally, *N*=*N*_*apo*_+*N*_*arr*_ is the total number of output factors. In both Eq. (5) and (6), the symbol $\sum $ stands for the average of the factors. The theoretical efficacy metric as a whole measures the influence of a particular node on both cell death and cell cycle arrest.

### Simulation results

Each GBM cell line has different genetic mutations. We consider 9 GBM cell lines, and their corresponding cellular mutations are given in Table 3. This information has been obtained using the GDSC database [12].

**Table 3:** GBM cell lines with different mutations.

Cell line name	Genetic mutations	Fault type (stuck-at 0/1)
42-MG-BA	p16INK4a, p14ARF, PDGFR, PTEN, p53	0,0,1,0,0
A172	p16INK4a, p14ARF, EGFR, p53	0,0,1,0
AM-38	p16INK4a, p14ARF, BRAF	0,0,1
CCF-STTG1	EGFR, MDM2, PTEN	1,1,0
LN-229	FasL, p16INK4a, p14ARF, EGFR,Grb2	0,0,0,1,1
T98G	FasL, p16INK4a, p14ARF, p53	0,0,0,0
U-87-MG	FasL, p16INK4a, p14ARF, PTEN, NF1	0,0,0,0,0
YKG-1	p16INK4a, p14ARF, p53, PTEN, PI3K, NF1	0,0,0,0,1,0

The set of input conditions should reflect that the stemness and growth pathways are active in the cancer cells, and the immune system has started to respond to cancer. The stemness-related genes Shh and Wnt are responsible for activating the stemness pathways, as can be seen in Fig. 4; we can also see how the growth factors GF contribute to tumor cell survival. The immune system response is controlled by cytokines, including IL17, TNF, and TGFb, as shown in Figs. 5 and 10. The tumor suppressor NF1 is active for the purpose of controlling tumor growth.

For this purpose, the input vector for the simulations has been assigned the value [1,1,1,1,1,1,0,1,0,0,0,0,0,0,0,0,0,0,0,0,0,1]; this implies that Shh, Wnt, GF, IL17, Cytokine, TNF, TGFb and NF1 are all set to one and the other inputs are set to zero. The other inputs such as Dopamine, GABA, Estrogen and other hormones do not have a direct effect on cell death or arrest; the genes downstream of these inputs interact with the cancer-related pathways, but do not play a significant role in glioblastoma development. Note that our prioritization method ranks each gene based on its effect on the output metric, irrespective of whether the upstream input is active or inactive.

In order to eliminate any bias based on the choice of inputs on drug prioritization, we normalize the theoretical efficacy by the ’NO FAULT NO DRUG’ value. The ’NO DRUG NO FAULT’ value is the value of fault free apoptosis and arrest; it is a number that captures the theoretical value of cell death and arrest in the absence of drugs or mutations. In the presence of mutations, such as in U-87-MG, the output value of the network falls to 1.3, which is lesser than the fault free value. For our chosen set of inputs, the ’NO DRUG NO FAULT’ value is 2.25, and we divide the value of the theoretical efficacy of a drug by 2.25 in order to measure to what degree can the drug restore the output of the network to its fault free value.

### Prioritization of genetic targets for the GBM cell line u-87 MG

We will demonstrate the results of our ranking technique given the genetic profile of a cell line. For example, in GBM cell line ’U-87 MG’, there are 5 faults in the cell line, and the corresponding faults vector is shown in Eq. (8). All the faults in this fault vector are stuck-at-0 faults. We shall pass the value [0,0,0,0,0] forcing the relevant nodes to zero, which means that all the 5 faults are active and that FasL, p16INK4a, p14ARF, PTEN, and NF1 are all down-regulated.

8 $$\begin{array}{@{}rcl@{}}  \text{Fault}& = [\mathrm{FasL, p16INK4a, p14ARF, PTEN, NF1}] \end{array} $$

**Fig. 12:**
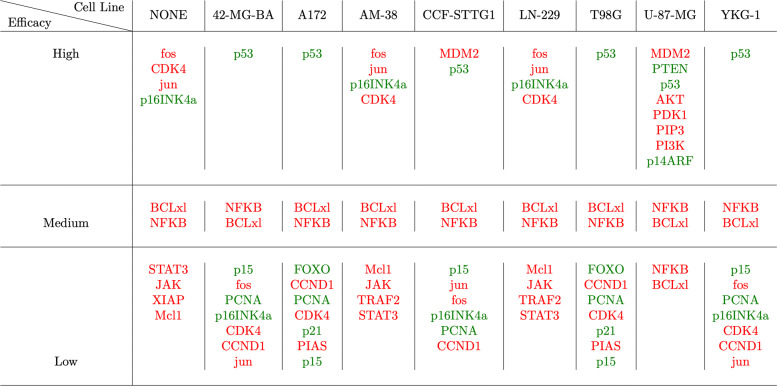
Prioritization of single targets. Green color represents the nodes that need to be activated and red color represent the nodes that need to be inhibited. LaTeX package tikz version 3.14 is used is used to render this figure

**Fig. 13:**
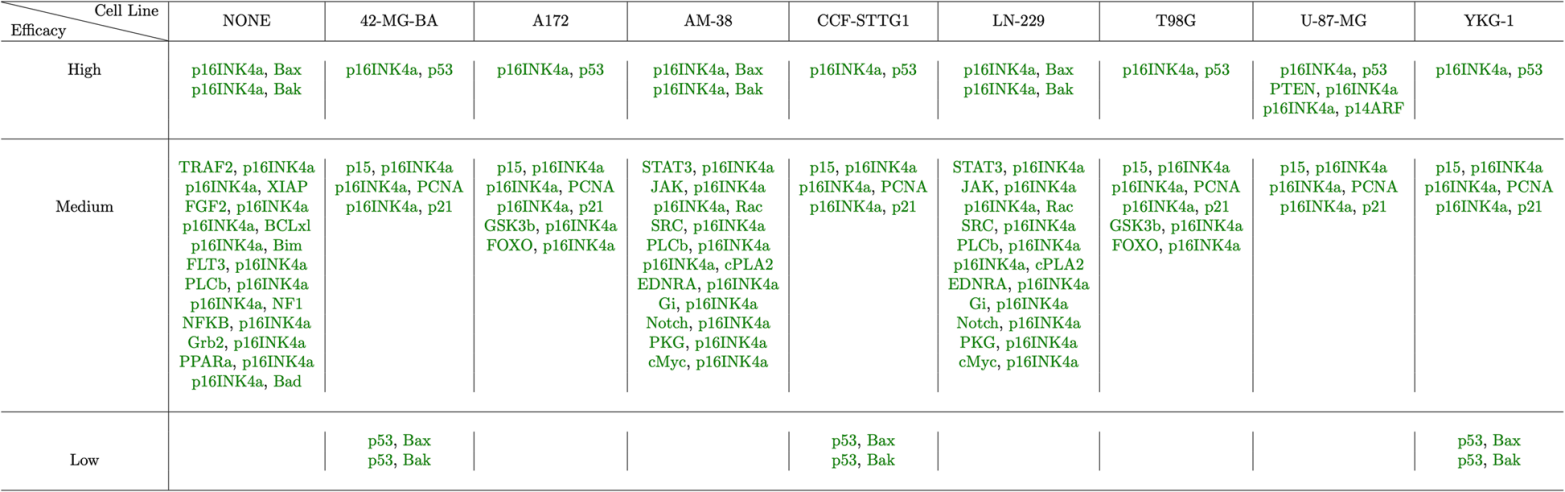
Prioritization of pairs of targets. Green color represents the nodes that need to be activated and red color represent the nodes that need to be inhibited. LaTeX package tikz version 3.14 is used is used to render this figure

Similarly, the fault vector for each cell line, given in Table 3, is passed one at a time to the Boolean network and we produce a ranking of the best targets to attack the tumor growth for that mutation profile. Figure 12 shows the results of prioritization for single genetic targets; ’High’ implies greater priority of the target. For instance for the cell line CCF-STTG1, MDM2 and p53 are two targets that have the same priority as each other, but have higher rank than NFKB and BCLxL. In Fig. 12, red text implies that the target should be inhibited and green text implies that a target should be expressed. We can use these results to find drugs that act on these genetic targets and have the desired action on those targets. For example, if we want to design a single target therapy with the best efficacy for a patient with genetic mutations similar to YKG-1, we should look for a drug that activates p53. The prioritization of pairs of targets is shown in Fig. 13.

### Drug sensitivity for anti-cancer and non-cancer drugs

**Fig. 14:**
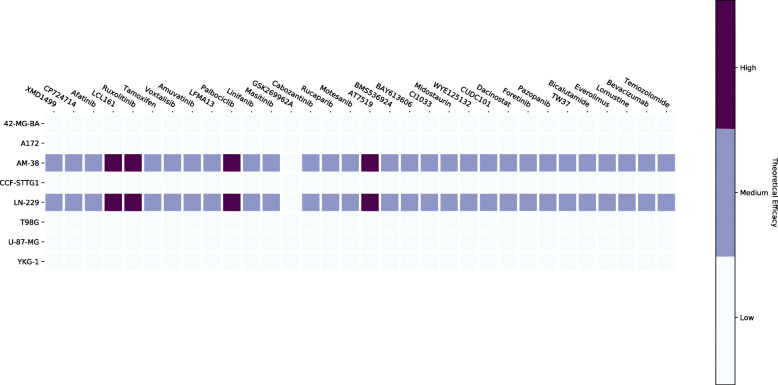
Drug Sensitivity for Anti-Cancer Drugs. Python package Matplotlib version 3.1.1 is used is used to render this figure

**Fig. 15:**
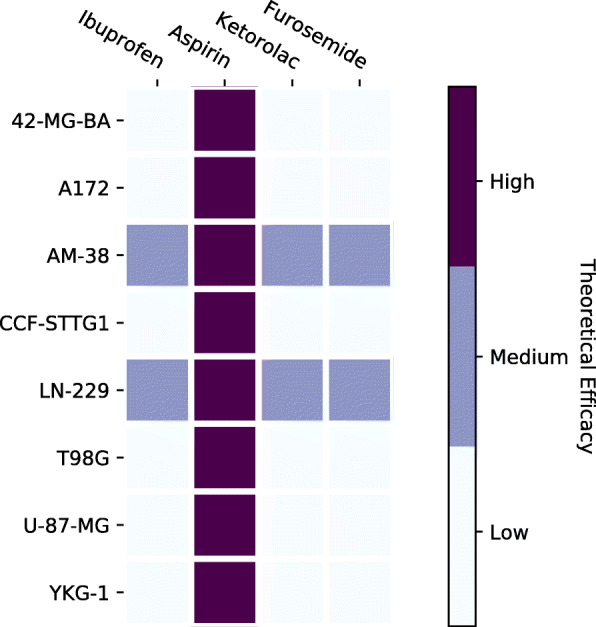
Drug Sensitivity for Non-Cancer Drugs. Python package Matplotlib version 3.1.1 is used is used to render this figure

The second simulation is run to test drug sensitivity for each different GBM cell line. The data for the drugs and their targets is from the GDSC database and DrugBank [12, 22]. Additional file 1: Table S5 shows the drug with its corresponding targets. Figure 14 shows the drug sensitivity for anti-cancer drugs as predicted by the Boolean model; each row corresponds to a GBM cancer cell line, and each column is a drug. Figure 15 shows the drug sensitivity for non-cancer drugs as predicted by the Boolean model. In both Figs. 14 and 15, a white cell implies that the drug does not work on that particular cell line, and a purple cell stands for a drug with high efficacy. We can see that Aspirin seems to work on many GBM cell lines. Light blue cells are the drugs that do not cause a significant change in the value of the theoretical efficacy metric. Temozolomide, in Fig. 14, does not have much effect on any of the GBM cell lines except AM-38 or LN-229; this might indicate that the other cell lines have developed resistance to TMZ.

### Increasing sensitivity to temozolomide

**Fig. 16:**
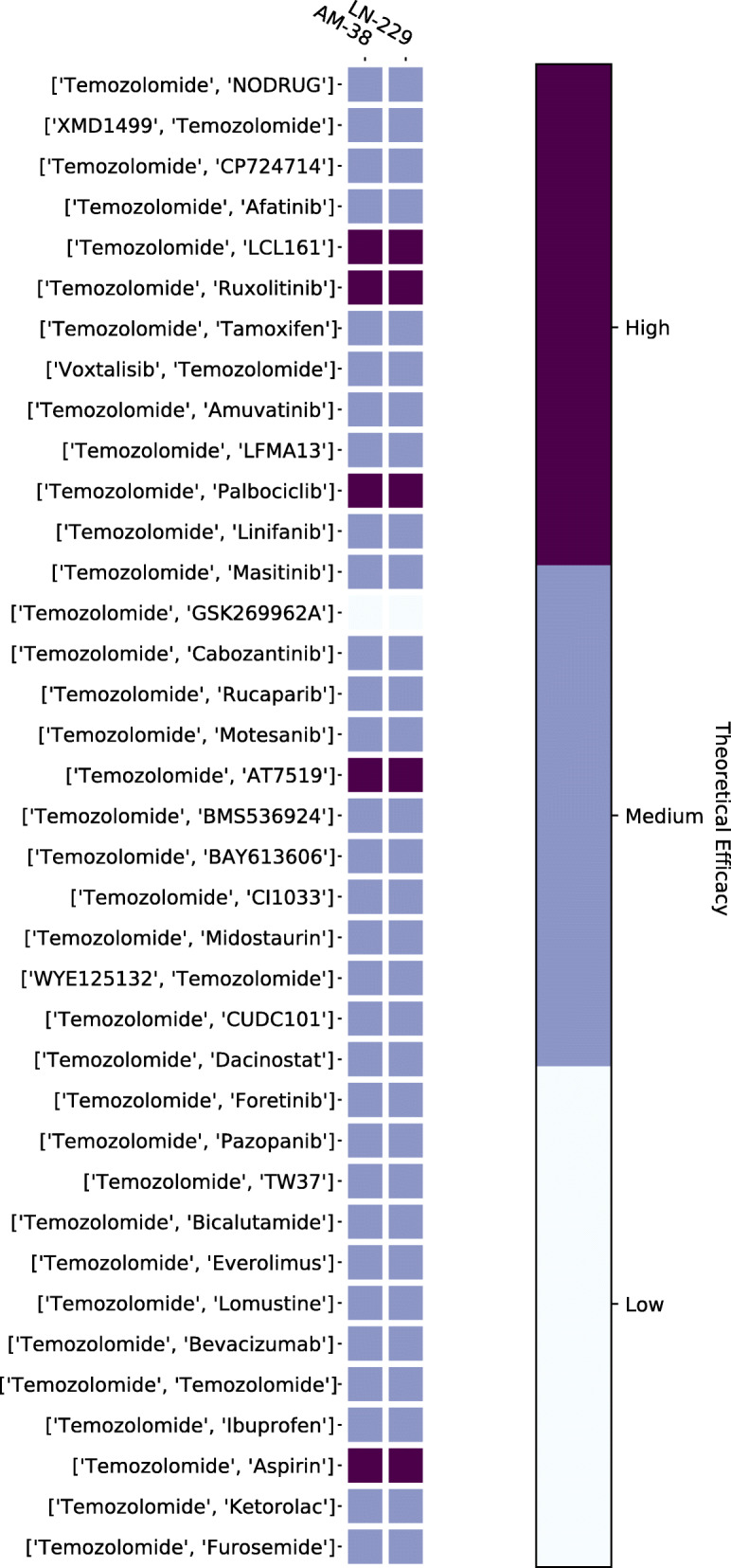
Increasing Sensitivity to Temozolomide. Python package Matplotlib version 3.1.1 is used to render this figure

We ran a simulation to test whether it is possible to reduce the resistance to TMZ in GBM cell lines. Figure 16 shows that only the combination of Aspirin and TMZ is able to increase the sensitivity of the cancer cells to TMZ in all of the cell lines, but the rest of the drugs seem to be unable to have a significant effect. Cell lines AM-38 and LN-229 have a slightly greater sensitivity to TMZ than the other cell lines. This tells us that while treating a patient with a genetic profile similar to any of the other cell lines we have considered, we might need to look at other two-drug or multi-drug therapies.

### Best two-drug combinations for GBM treatment

We can predict the best two-drug combination of anti-cancer and non-cancer drugs that can work for the LN-229 and AM-38 GBM cell lines. We chose these two cell lines since they are sensitive to most of the drugs in our analysis, as we saw in Fig. 14. Table 4 shows only the top 10 two-drug combinations. The best two combinations are Aspirin + Palbociclib and Aspirin + AT7519; both these combinations have equal efficacy and perform 2*%* better than the next best combination. It is interesting to note that Aspirin features in the top 10 combinations, it is a non-cancer drug and not usually considered while designing drug therapies for GBM treatment.

**Table 4:** Best performing two-drug combinations

Rank	Combination
1	Palbociclib, Aspirin
2	Aspirin, AT7519
3	Palbociclib, LCL161
4	LCL161, AT7519
5	Ruxolitinib, Palbociclib
6	Ruxolitinib, AT7519
7	Palbociclib, NODRUG
8	NODRUG, AT7519
9	XMD1499, Palbociclib]
10	XMD1499, AT7519

## Discussions

### Prioritization of genetic targets for any GBM cell line

We employed our prioritization technique to predict most effective targets for the treatment of the GBM cell lines. Using this functionality, we could move towards a personalized medicine approach. The patient’s genetic mutations could be fed into the algorithm as faults, and we could perform the prioritization task to identify the key intervention points specifically effective for that patient. We could use the prioritization results to design new drugs or drug therapies to treat GBM.

We can predict the effectiveness of a *N*-target treatment using the prioritization method. If we rank all possible combinations of targets on the same scale, it is possible to determine the optimal number of intervention points for treatment of GBM.

### Drug sensitivity for anti-cancer and non-cancer drugs

We compared the theoretical efficacy for several anti-cancer and non-cancer drugs. This functionality is similar to the one available in GDSC. Additionally, we performed the sensitivity analysis for non-cancer drugs. It is was interesting to see that non-cancer drugs could potentially be effective in killing or stopping the growth of cancer cells.

### Best two-drug combinations for GBM treatment

We ranked all possible two-drug combinations for the given set of anti-cancer and non-cancer drugs. This functionality could be extended to test the combination of *n* number of drugs and then to find the optimal drug combination (from the existing drugs available in the market) customized to the patient’s genetic profile.

## Conclusion

We modeled the biological pathways instrumental in Glioblastoma Multiforme and identified drug therapies that could prove to be effective for GBM treatment. We predicted a prioritization of genetic targets given the genetic profile of a patient. The Boolean model predicts that Aspirin, a non-cancer drug, could potentially reduce the resistance to Temozolomide in GBM patients. It could also be effective in combination with other chemotherapeutic drugs. Finally, we predicted two-drug therapies that could be more successful than the currently used treatment strategies.

The result of this study in drug combination ranking can be utilized in future GBM therapeutic experimental research. It was shown that the combination of Aspirin and TMZ can have a significant improvement in our desired outcome to increase the efficacy of GBM treatment efficacy. Several observational and biological studies have shown the anti-cancer effects of Aspirin for different cancers including glioblastoma [23–25]. It is shown that Aspirin can induce cell cycle arrest [26] which is consistent with our findings here. Therefore, our model not only confirms the benefits of Aspirin in GBM treatment but it also provides strategies of how to effectively use Asprin in drug combination therapies.

## Supplementary Information

**Additional file 1** **Table S5**: Targets of cancer and anti-cancer drugs.

## Data Availability

The datasets generated during and/or analysed during the current study are available in the GitHub repository, https://github.com/sarafradhika/Glioblastoma_Boolean_Network. Declarations

## Abbreviations

FDA: Food and drug administration
GBM: Glioblastoma multiforme
IDH: Isocitrate dehydrogenase
MGMT: Methylguanine-DNA methyltransferase
mRNA: messenger RNA
SOC: Standard of care
STAT3: Signal transducer and activator of transciption 3
TMZ: Temozolomide
