# Supplementary material for: Drug target ranking for glioblastoma multiforme
Source: BMC Biomed Eng. 2021 Apr 26;3:7. doi: 10.1186/s42490-021-00052-w (PMC8074458; doi:10.1186/s42490-021-00052-w)
Supplement: Supplementary file 1 — Additional file 1 Table S5: Targets of cancer and anti-cancer drugs. [file 42490_2021_52_MOESM1_ESM.pdf]

Number	Drug	Genetic Target
1	XMD1499	ALK, CDK4
2	CP724714	ERBB2
3	Afatinib	ERBB2, EGFR
4	LCL161	XIAP
5	Ruxolitinib	JAK1
6	Tamoxifen	ESR1
7	Voxtalisib	PI3K, MTOR
8	Amuvatinib	KIT, PDGFR, FLT3
9	LFMA13	BTK
10	Palbociclib	CDK4
11	Linifanib	VEGF, KDR, FLT3, FLT4, KIT
12	Masitinib	KIT, PDGFR
13	GSK269962A	ROCK
14	Cabozantinib	KDR, MET, KIT, FLT1, FLT3, FLT4
15	Rucaparib	PARP1
16	Motesanib	KDR, KIT, PDGFR
17	AT7519	CDK4
18	BMS536924	IGF1R
19	BAY613606	SYK
20	CI1033	EGFR, ERBB2
21	Midostaurin	PKC,FLT1
22	WYE125132	mTOR
23	CUDC101	HDAC1, EGFR, ERBB2
24	Dacinostat	HDAC1
25	Foretinib	MET, KDR, FLT4, PDGFR, FGF2, EGFR
26	Pazopanib	KIT, PDGFR
27	TW37	BCL2, BCL-XL, MCL1
28	Bicalutamide	AR
29	Everolimus	MTOR
30	Lomustine	IDH1, MGMT
31	Bevacizumab	FasL
32	Temozolomide	OG
33	Ibuprofen	THBD, Gp1BA, COX2, PPARa, PPARg, BCL2
34	Aspirin	EDNRA, ERK, NFKB, AMPK, RSK2, COX2, cMYC, p53, PCNA
35	Ketorolac	COX2
36	Furosemide	CA
